# A comprehensive characterization of aggravated aging-related changes in T lymphocytes and monocytes in end-stage renal disease: the iESRD study

**DOI:** 10.1186/s12979-018-0131-x

**Published:** 2018-11-08

**Authors:** Yen-Ling Chiu, Kai-Hsiang Shu, Feng-Jung Yang, Tzu-Ying Chou, Ping-Min Chen, Fang-Yun Lay, Szu-Yu Pan, Cheng-Jui Lin, Nicolle H R Litjens, Michiel G H Betjes, Selma Bermudez, Kung-Chi Kao, Jean-San Chia, George Wang, Yu-Sen Peng, Yi-Fang Chuang

**Affiliations:** 1 Division of Nephrology, Department of Medicine, Far Eastern Memorial Hospital, Taipei, Taiwan; 2 Graduate Institute of Clinical Medicine, College of Medicine, National Taiwan University , Taipei, Taiwan; 3 Graduate Program in Biomedical Informatics, Yuan Ze University, Taoyuan, Taiwan; 4 Graduate Institute of Immunology, College of Medicine, National Taiwan University , Taipei, Taiwan; 5 Department of Medicine, National Taiwan University Hospital Yun Lin Branch, Douliu, Taiwan; 6 Division of Nephrology, Department of Internal Medicine, Mackay Memorial Hospital, Taipei, Taiwan; 7 Department of Internal Medicine, Nephrology and Transplantation, Erasmus Medical Center, University Medical Center Rotterdam, Rotterdam, Netherlands; 8 International Health Program, National Yang Ming University School of Public Health, Taipei, Taiwan; 9 Biology of Healthy Aging Program, Division of Geriatric Medicine and Gerontology, Johns Hopkins University School of Medicine, Baltimore, MD USA; 10 Institute of Public Health, National Yang Ming University School of Public Health, Taipei, Taiwan; 11 Preventive Medicine Research Center, National Yang-Ming University, Taipei, Taiwan

**Keywords:** Immunosenescence, Aging, CVD, Inflammation, ESRD

## Abstract

**Background:**

Patients with end-stage renal disease (ESRD) exhibit a premature aging phenotype of the immune system. Nevertheless, the etiology and impact of these changes in ESRD patients remain unknown.

**Results:**

Compared to healthy individuals, ESRD patients exhibit accelerated immunosenescence in both T cell and monocyte compartments, characterized by a dramatic reduction in naïve CD4+ and CD8+ T cell numbers but increase in CD8+ T_EMRA_ cell and proinflammatory monocyte numbers. Notably, within ESRD patients, aging-related immune changes positively correlated not only with increasing age but also with longer dialysis vintage. In multivariable-adjusted logistic regression models, the combination of high terminally differentiated CD8+ T cell level and high intermediate monocyte level, as a composite predictive immunophenotype, was independently associated with prevalent coronary artery disease as well as cardiovascular disease, after adjustment for age, sex, systemic inflammation and presence of diabetes. Levels of terminally differentiated CD8+ T cells also positively correlated with the level of uremic toxin *p*-cresyl sulfate.

**Conclusions:**

Aging-associated adaptive and innate immune changes are aggravated in ESRD and are associated with cardiovascular diseases. For the first time, our study demonstrates the potential link between immunosenescence in ESRD and duration of exposure to the uremic milieu.

**Electronic supplementary material:**

The online version of this article (10.1186/s12979-018-0131-x) contains supplementary material, which is available to authorized users.

## Background

Patients with end-stage renal disease (ESRD) exhibit many physiological changes reminiscent of accelerated aging processes and have an increased mortality and susceptibility to diseases when compared to chronological age-matched individuals [1]. Impaired physical functions, muscle wasting, cognitive function decline, accelerated vascular disease and increased risks of death are among the many aging-related complications increased in frequency in ESRD [2]. The immune system of ESRD patients also exhibits significant changes from that of healthy individuals. For example, while a low grade-inflammation can be observed during normal aging [3], it is significantly enhanced by uremia [4]. Accompanying low-grade inflammation, immune cells develop different phenotypic markers and functions during normal aging. These changes are are collectively called “immunosenescence” and are considered to contribute to various aging-related morbidities, including increased risks for infectious events and cardiovascular diseases [5–7].

During normal aging, lymphocytes and monocytes experience dramatic changes. The subset distribution in the CD8+ T cell compartment is different between young and old people; with progressive terminal differentiation [8], loss of co-stimulatory molecules, shortening of telomeres and impaired response toward infectious pathogens and vaccinations [9, 10] occur during aging. CD4+ T cells also exhibit aging-related changes. For example, naïve CD4 T cells from aged animals show reduced IL-2 production, proliferation, helper function, effector generation and memory function [11]. Premature aging of the T cell compartment has been observed in ESRD patients, characterized by decreased thymic output of naïve cells and increased susceptibility toward apoptosis [12]. We had previously reported that higher levels of CD4+ CD28- cells can be found in ESRD patients [13] and CD4+ T cells activation is affected in ESRD patients in an age-dependent manner [14]. Recently, it has been reported that elderly kidney transplant patients also exhibit more advanced T cell differentiation compared to younger patients [15]. Besides lymphocytes, CD14++CD16+ intermediate monocytes as well as the CD14+ CD16++ non-classical monocytes also increase in numbers during aging [16] and are further increased in ESRD patients [17]. CD14++CD16+ intermediate monocytes are of particular interest because these cells produce high levels of TNF-α and IL-6 upon activation and are involved in many infectious and pathogenic inflammatory diseases [18, 19].

As a result, enhanced aging-related immune changes can be considered as one characteristic of the premature aging phenotype of renal failure. However, it remains unclear how mechanistically the immune system suffers from these enhanced aging-related changes in renal failure patients. In addition, previous studies attempted to characterize the immune system of ESRD patients were frequently based on small numbers of patient and did not include both monocyte and lymphocyte panels at the same time. We hypothesize that if ESRD patients have accelerated aging, exposure of immune cells to the uremic milieu will also have an impact on immunosenescence, independent of chronological age. In addition, profiling both adaptive and innate immune subsets should be performed simultaneously to better understand the overall effects of uremia on aging-related immune responses.

We thus initiated the *Immunity in ESRD study*, or the iESRD study to comprehensively characterize the immune changes in ESRD. The iESRD study is an on-going longitudinal cohort study with the ultimate goal to investigate if immune changes are associated with long-term clinical outcomes. Here, we present the findings from analyzing the baseline data of this study based on 412 hemodialysis patients.

## Methods

### Study participants

The immunity in ESRD study (iESRD) is a multicenter study which recruited ESRD patients undergoing regular hemodialysis with age > 20 years at two academic teaching hospitals in Taiwan: the Far Eastern Memorial Hospital and the National Taiwan University Hospital Yun Lin branch. A total 432 patients signed informed consent and were screened for eligibility. Those with recent hospitalization within three months, active infection, incomplete blood test results or poor blood samples quality were excluded, making only 412 patients included in the final study (198 from Far Eastern Memorial Hospital and 214 from National Taiwan University Hospital).

CMV serostatus in all individuals were determined using the Roche Elecsys assay. All methods were carried out in accordance with relevant guidelines and regulations. All experimental protocols were approved by the Research Ethics Committee of both institutions (FEMH 103084-E and NTUYL 201511092 RINA). Informed consent was obtained from all participants and/or their legal guardians.

### Data collection

Biochemical data were collected on the same day of peripheral blood mononuclear cells (PBMCs) sampling. Blood samples were collected before start of a hemodialysis session in the middle of week. Diagnosis of coronary artery disease (CAD) was defined as either 1) > 50% stenosis of at least one coronary artery on coronary angiography or 2) documented reperfusion defect on stressed nuclear medicine scan. Peripheral arterial occlusive disease and stroke were based on medical chart review. Cardiovascular disease (CVD) is defined by the medical history of either CAD, peripheral arterial occlusive disease or stroke.

### T cell and monocyte differentiation panel

On the day of blood sampling, PBMCs were isolated by Ficoll-Paque PLUS gradient centrifugation following the manufacturer’s protocol (GE Healthcare). Freshly isolated PBMCs were immediately stained with antibody cocktails and processed for flow cytometer reading and analysis as previously described [12, 17]. The gating strategy is shown in Additional file 1: Figure S1. Briefly, singlets were identified by forward scatter area and height. Lymphocytes were subsequently gated by forward and side scatter characteristics, and anti-CD3-AF700 (clone UCHT1, Biolegend) was used to identify CD3+ T cells. CD4+ and CD8+ T cells, determined by anti-CD4-PerCP-Cy5.5 (clone OKT4, Biolegend) and anti-CD8-APC-Cy7 (clone SK1, Biolegend), were further analyzed by their surface anti-CCR7-APC (clone G043H7, Biolegend) and anti-CD45RA-Alexa488 (clone HI100, Biolegend) expression to separate into the CCR7+ CD45RA+ T_NAIVE_ subset, the CCR7+ CD45RA-T_CM_ subset, the CCR7-CD45RA-T_EM_ subset and the CCR7-CD45RA+T_EMRA_ subset. CD28-PE-Cy5 (clone CD28.2, eBioscience) and CD95-PE (clone DX2, eBioscience) were used to further define stem cell memory T cells (Tscm) from the T_NAIVE_ subset.

Anti-CD86-PE (clone IT2.2, eBioscience) was used to gate the CD86+ monocytes. By anti-CD14-FITC (clone M5E2, Biolegend) and anti-CD16-APC (clone 3G8, eBioscience), monocytes were further classified as classical (Mon1, CD14++CD16-), intermediate (Mon2, CD14++CD16+), and non-classical (Mon3, CD14 + CD16++) monocytes. We used a clinical complete blood count (CBC) analyzer Beckman Coulter LH 780 to determine the absolute lymphocyte and monocyte counts for each sample and subset cell numbers were subsequently enumerated. All the experiments were performed in Far Eastern Memorial Hospital and analyzed using a Beckman Coulter MoFlo™-XDP multicolor flow cytometer.

### Measurements of uremic toxin *p*-cresyl sulfate and indoxyl sulfate

Serum *p*-cresyl sulfate (PCS) and indoxyl sulfate (IS) were measured with liquid chromatography-mass spectrometry (4000 QTRAP, USA). In brief, serum samples were prepared and deproteinised by heat denaturation. The concentrations of IS and PCS were measured in serum ultrafiltrates, obtained by using Microcon YM-30 separators (Millipore, Billerica, MA, USA). HPLC was performed at room temperature using a dC18 column (3.0 × 50 mm, Atlantis, Waters). The sensitivity of this assay was 1 μg/L for PCS and 1 μg/L for IS.

### Statistical analyses

Baseline characteristics were described as mean ± standard deviation for continuous variables, and frequency for categorical variables. Spearman’s correlation was applied to evaluate the correlation of immunological markers with age and biochemical data. Partial regression plots were used to analyze the relationships between immune cell subset percentages and age adjusting for dialysis vintage, or between immune cell subset percentages and dialysis vintage adjusting for age. CAD and CVD were analyzed separately as in most cardiovascular outcome studies.

The R corrplot package was used to draw the correlogram to visualize the relationships between immune cell subsets (Freely available at http://www.sthda.com/english/wiki/visualize-correlation-matrix-using-correlogram). A *p* value of more than 0.05 was considered insignificant and only significant results are shown on the correlogram.

Logistic regression models, adjusted for age, gender, albumin, hemoglobin, diabetes mellitus, and hs-CRP, were used to evaluate the independent relationship between immunophenotype and the presence of CAD or CVD. All statistical tests were two-tailed, and a *p* value of less than 0.05 was considered be significant. The statistical analyses were performed with STATA version 13.1.

## Results

### Aggravated aging-related immune changes in ESRD patients

First, we compared the immune cell subsets in the peripheral blood between 412 ESRD patients and 57 age-matched healthy individuals using multicolor flow cytometry (representative staining, Additional file 1: Figure S1). The demographic and biochemical data of the iESRD participants are summarized in Table 1. Main causes of ESRD were diabetes (37.3%), chronic glomerulonephritis (27.6%), hypertension (14.3%) and others (20.8%). Because cytomegalovirus (CMV) infection profoundly affects human immune system homeostasis, we first tested CMV seropositivity frequency among participants. All healthy individuals (*n* = 57; 100% CMV seropositive) were CMV seropositive and only 4 out of 412 ESRD patients were seronegative for CMV (99% seropositive). Despite the majority of our study samples was CMV seropositive, we detected many immune subsets differences between healthy versus ESRD (Table 2). For both CD4+ and CD8+ T cells, ESRD patients demonstrated lower percentages of T_NAIVE_ cells but increased percentages of memory stem T_SCM_ cells, which are the considered to be the least differentiated memory T cells in humans and plays an important role in immune protection upon pathogen rechallenge [20]. Interestingly, these antigen-experienced, naïve phenotype T cells recently were reported to increase during aging [21]. CD8+ Effector memory T_EM_ and terminally differentiated T_EMRA_ cells, both memory T cells with higher levels of differentiation, were increased in percentages in ESRD patients. Besides these distributional changes, ESRD patients exhibit a dramatic 40–50% reduction in CD4+ and CD8+ naïve T cell numbers and especially have a significant increase in their CD8+ T_EMRA_ cell numbers. For CD4+ T cells, although percentages of T_EM_ and T_EMRA_ subsets were not significantly increased, the absolute cell number of T_EM_ cells was increased in ESRD patients.

Significant differences in the monocyte differentiation status were also found (Table 2). ESRD patients exhibited higher percentages of intermediate and non-classical monocytes and lower percentages of classical monocytes in their peripheral blood. In absolute cell number terms, the intermediate and non-classical monocytes were both significantly increased. Similar to T_EMRA_ cells, levels of intermediate monocytes and non-classical monocytes are known to increase during aging [16]. Overall, our observations confirmed that many immunological changes observed in ESRD are reminiscent of immunosenescence observed during normal aging.

We next tested whether T cell compartment changes and monocyte compartment changes are related. As shown in Additional file 1: Figure S2, we performed correlogram analyses in both healthy and ESRD individuals using either cell type percentage or absolute cell numbers. We found that monocyte subset distribution and T cell differentiation are not significantly correlated, but cells of the same lineage tend to be significantly correlated in absolute number.

**Table 1:** Demographic data of iESRD participants

Variable	Mean (SD)
Age (years)	61.7 (12.2)
Male (%)	50.7
Diabetes (%)	44.6
Malignancy (%)	12.1
Dialysis vintage (years)	6.2 (5.1)
Albumin (g/dL)	4.0 (0.4)
Hemoglobin (g/dL)	10.9 (1.4)
T-cholesterol (mg/dL)	152.2 (37.3)
Triglyceride (mg/dL)	147.1 (95.4)
intact-PTH (pg/mL)	374.5 (423.6)
Calcium (mg/dL)	9.3 (0.8)
Phosphate (mg/dL)	4.9 (1.4)
Kt/V (Gotch)	1.4 (0.2)

The complete demographic data of 412 iESRD participants is shown

**Table 2:** Comparisons of immune cell subsets between ESRD and controls

Cell subset percentage	Healthy (57)	ESRD (412)	*P* value
CD4+ T cells	62.8 (10.3)	56.8 (13.3)↓	0.001*
Naïve T cells	41.6 (15.6)	28.5 (12.9)↓	< 0.001*
Stem Memory T cells	3.18 (2.01)	7.50 (6.24)↑	< 0.001*
Central Memory T cells	30.7 (9.6)	41.6 (11.1)↑	< 0.001*
Effector Memory T cells	27.0 (14.7)	28.3 (12.9)	0.47
Terminally Differentiated T cells	1.80 (2.24)	2.36 (2.72)	0.13
CD8+ T cells	26.5 (8.97)	29.2 (10.1)	0.051
Naïve T cells	32.9 (16.6)	21.8 (16.1)↓	< 0.001*
Stem Memory T cells	4.78 (5.26)	7.66 (6.20)↑	0.002*
Central Memory T cells	6.30 (3.58)	7.02 (7.91)	0.50
Effector Memory T cells	29.1 (11.7)	34.1 (16.6)↑	0.023*
Terminally Differentiated T cells	32.9 (14.4)	38.1 (16.7)↑	0.025*
Monocytes			
Classical Monocytes	64.1 (12.7)	56.9 (11.7)↓	< 0.001*
Intermediate Monocytes	6.25 (4.91)	10.1 (6.55)↑	< 0.001*
Non-Classical Monocytes	14.1 (10.8)	19.9 (9.7)↑	< 0.001*
Absolute cell number	Healthy (57)	ESRD (412)	*P* value
CD4+ T cells	530 (307)	523 (232)↓	0.02*
Naïve T cells	247 (199)	164 (112)↓	< 0.001*
Stem Memory T cells	17.2 (15.5)	11.5 (9.1)↓	< 0.001*
Central Memory T cells	188 (114)	229 (116)	0.65
Effector Memory T cells	89.0 (49.5)	120 (86.4)↑	0.006*
Terminally Differentiated T cells	5.76 (7.59)	9.25 (11.7)	0.10
CD8+ T cells	277 (270)	275 (180)↓	0.012*
Naïve T cells	103 (97.7)	54.5 (61.9)↓	< 0.001*
Stem Memory T cells	13.7 (17.6)	4.63 (5.50)↓	< 0.001*
Central Memory T cells	11.6 (9.22)	12.1 (13.9)	0.47
Effector Memory T cells	92.9 (58.0)	102 (83.6)	0.26
Terminally Differentiated T cells	70.2 (53.9)	105 (95.2)↑	0.013*
Monocytes			
Classical Monocytes	248 (91.2)	264 (141)	0.13
Intermediate Monocytes	19.2 (21.9)	40.3 (33.9)↑	0.001*
Non-Classical Monocytes	18.4 (12.0)	56.3 (38.2)↑	< 0.001*

Percentages and absolute numbers (per μl blood) of naïve (T_NAIVE_), stem cell memory (T_SCM_), central memory (T_CM_), effector memory (T_EM_), terminally differentiated (T_EMRA_) subsets and three monocyte subsets (classical monocytes, intermediate monocytes, non-classical monocytes) were shown as mean (SD) and were compared between healthy controls and ESRD patients. The inter-group differences were analyzed by Student’s *t*-test

*P value < 0.05

### Dialysis vintage positively associates with immunosenescence

**Fig. 1:**
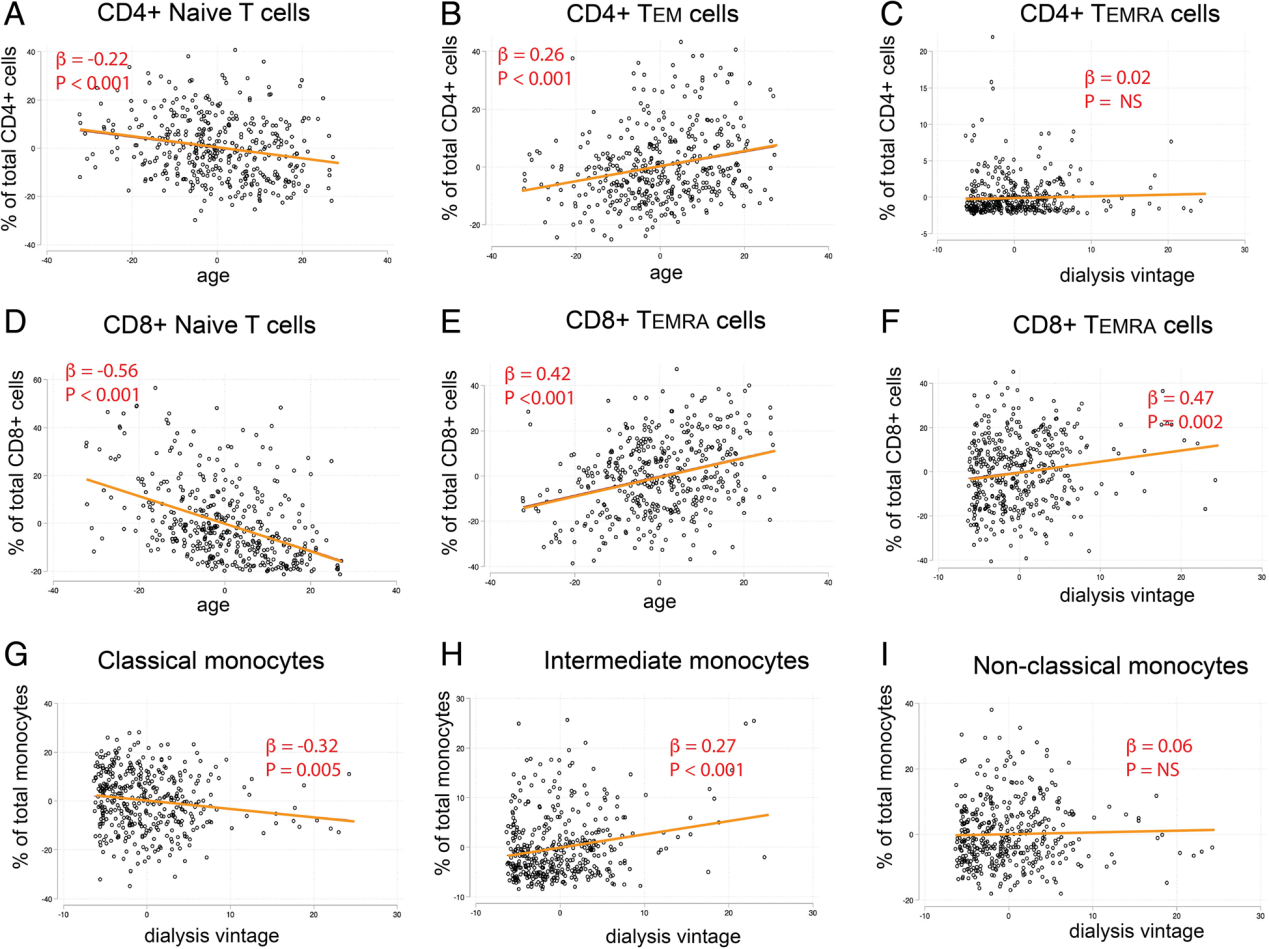
Independent associations between immune cell percentages with age and dialysis vintage. Scatter plots and regression lines demonstrated the relationship between immune cell differentiations with age or dialysis vintage in ESRD patients. Since dialysis vintage potentially modulates the effects of age on immunophenotype, we used partial regression plots to show the relationship between immune cell subset percentage and age adjusting for dialysis vintage, or between immune subset percentage and dialysis vintage adjusting for age. When indicated, the Y axis presents residuals from regressing immune cell subset percentage against dialysis vintage or age while the X axis presents residuals from regressing age against dialysis vintage or dialysis vintage against age. For presentation, the axes were labeled as they are instead of e(age|X) or e(vintage|X)

Although ESRD patients clearly exhibit aggravated immune aging, the etiology of aggravated immune aging remains unclear. We hypothesize, if the uremia milieu affects immune cell homeostasis, duration of ESRD or dialysis treatment (dialysis vintage years) should have a significant impact on the severity of observed aging phenotype, independent from the effect of age. We next interrogated the relationship between percentage of each immune cell subset with age and dialysis vintage in multivariable-adjusted regression models. The complete regression analysis results are shown in Table 3 and key representative plots are shown in Fig. 1. Because longer dialysis vintage was associated with the progressive decrease in total T cell counts (significant for both CD4+ and CD8+ T cells, data not shown), for this analysis we used subset percentages to reflect premature aging of each cell compartment instead of absolute cell counts. As shown in Table 3, age profoundly affected the T cell differentiation status by decreasing the percentage of T_NAIVE_ cells and increasing the percentage of T_EM_ and T_EMRA_ cells. Both CD4+ and CD8+ T_NAIVE_ cells decrease in percentage with aging, but effects of age on T_EMRA_ cells were more pronounced in the CD8+ compartment than CD4+ cells. Consistent with a previous study made in non-renal failure population [16], we also found that age positively associated with the percentage of intermediate monocytes. When we further adjusted etiology of ESRD in the model, the relationships between age and immune cell subsets did not change (data not shown). Longer dialysis vintage years robustly associated with higher percentages of CD8+ T_EMRA_ cells (β = 0.47, *p* = 0.002). Importantly, dialysis vintage also positively associated with percentages of intermediate monocytes and negatively associated with the percentages of classical monocytes. To further confirm the effects of dialysis vintage on immune changes, ESRD patients were separated into tertiles based on vintage years for trend analysis and were also analyzed by robust regression to eliminate the concern of outliers (Additional file 1: Table S1). CD4+ CD28null cells are important aging-related T cell subset that had been reported to increase during aging. Although CD4+ CD28null cells were also increased in dialysis patients, neither percentages nor absolute counts of CD4+ CD28null cells in dialysis patients were correlated with dialysis vintage (data not shown). Overall, the dialysis vintage, after statistical adjustment for age, significantly associated with both immunosenescent T cell differentiation (especially in CD8+ T cells) and higher levels of intermediate monocytes.

**Table 3:** Independent effects of age and dialysis vintage on immune cell aging

Cell Subset (percentage)	Age		Dialysis Vintage	
	β	*P* value	β	*P* value
CD4+ T cells				
Naïve T cells	−0.22	< 0.001*	0.20	0.095
Stem Memory T cells	0.08	0.029*	0.03	0.72
Central Memory T cells	−0.05	0.24	−0.60	< 0.001*
Effector Memory T cells	0.26	< 0.001*	0.41	0.001*
Terminally Differentiated T cells	0.02	0.12	0.02	0.46
CD8+ T cells				
Naïve T cells	−0.56	< 0.001*	0.14	0.31
Stem Memory T cells	0.12	0.002*	0.001	0.91
Central Memory T cells	−0.01	0.61	0.11	0.16
Effector Memory T cells	0.16	0.014*	−0.67	< 0.001*
Terminally Differentiated T cells	0.42	< 0.001*	0.47	0.002*
Monocytes				
Classical Monocytes	0.02	0.72	−0.32	0.005*
Intermediate Monocytes	−0.03	0.21	0.27	< 0.001*
Non-Classical Monocytes	−0.02	0.64	0.06	0.56

To separate the effects of age from dialysis vintage on immune changes, we tested the independent effects of age and dialysis vintage on cell subset percentages. In a multivariable-adjusted regression model (using subset percentage as the independent variable), the independent associations between immune cell percentage and age as well as the independent associations between immune cell percentage and vintage are shown

*P value < 0.05

### Aging-related immune changes correlate with cardiovascular risk factors and systemic inflammation

It is well-known that ESRD patients exhibit a dramatic increased risk for cardiovascular disease when compared to age-matched healthy individuals [22]. In the literature, inflammation is responsible for increased risk of atherosclerotic diseases and mortality in ESRD because ESRD patients also exhibit high level of chronic inflammation [23, 24]. Since immunosenescence contributes to atherosclerotic diseases in the elderly without renal diseases [25], we studied the correlation between parameters of immunosenescence with traditional as well as non-traditional cardiovascular risk factors in the iESRD cohort. We selected CD8+ T_NAIVE_, CD8+ T_EMRA_ and intermediate monocytes as key immunosenescence parameters to perform further analysis in the current study because both adaptive and innate immunity were implicated in previous studies on atherosclerotic vascular diseases [26] and these subsets were closely associated with age and/or dialysis vintage. As shown in Additional file 1: Table S2, these immune changes were associated with traditional and non-traditional cardiovascular risk factors. Most importantly, systemic inflammation as measured by high-sensitivity C-reactive protein was associated with decreased CD8+ T_NAIVE_ and increase in intermediate monocyte numbers. The presence of diabetes, another important cardiovascular risk factor, has little impact on the extent of immunosenescence (Additional file 1: Table S3).

### ESRD patients with concurrent cardiovascular disease display more severe immunosenescence

To test the impact of aging-related immune changes on cardiovascular health, percentages and cell numbers of CD8+ T_NAIVE_, CD8+ T_EMRA_ cells and intermediate monocytes were further compared between patients with and without coronary artery disease (CAD) and between patients with and without cardiovascular disease (CVD). Among 412 patients, 106 patients had history of coronary artery disease determined by history of myocardial infarction, positive coronary angiography or positive thallium scan; 132 patients had cardiovascular disease defined by the history of either coronary artery disease as defined in the method section, stroke, or peripheral arterial occlusive disease. As shown in Additional file 1: Table S4, patients with CAD or CVD had lower percentages of CD8+ naive T cells and higher percentages of CD8+ T_EMRA_ cells. Patients with CVD also had significant higher percentages of intermediate monocytes.

### The high-CD8^+^T_EMRA_/high-intermediate monocyte immunophenotype independently associates with existing cardiovascular diseases

Although patients with concurrent cardiovascular disease had higher percentages of CD8+ T_EMRA_ cells and CD14++CD16+ intermediate monocytes in their peripheral blood, the differences between groups were relatively small regarding a given immune subset. These findings prompted us to create a composite immunophenotype based on both cell subsets. To date, no study has studied both adaptive and innate immune cells simultaneously to investigate whether aging-related immune changes in ESRD patients (or even individuals with normal renal function) are related to atherosclerotic complications. To characterize a clinically useful phenotype based on both cell types, we tested the use of medium-split of each variable and defined immunophenotypes based on expression levels of CD8+ T_EMRA_ and intermediate monocyte. We found that all the iESRD participants can be separated into four groups: high CD8+ T_EMRA_/high intermediate monocytes, high CD8+ T_EMRA_/low intermediate monocytes, low CD8+ T_EMRA_/high intermediate monocytes and the low CD8+ T_EMRA_/low intermediate monocytes. We then tested the independent associations of immunophenotypes with the concomitant presence of CAD and CVD. We found that the “high CD8+ T_EMRA_/high intermediate monocyte” phenotype is independently associated with the presence of CAD and CVD (Table 4). We performed the likelihood ratio test to compare models with and without immunophenotype in the presence of age, DM, CRP and Hb, and the result was significant (*p* value = 0.0137). This suggests that immunophenotype as a whole results in a statistically significant improvement in model fit. When cell percentages instead of cell numbers were used in the model, the associations between immunophenotype and CAD or CVD remains statistically significant (Additional file 1: Table S5).

**Table 4:** A combinatorial aging-associated immunophenotype independently associates with coronary artery disease and cardiovascular disease

	OR (95% CI)	*P* value
Variables in model (independent variable: CAD)		
Immunophenotype		
High CD8+ T_EMRA_ High Mon_INT_	2.40 (1.18–4.90)	0.016*
High CD8+ T_EMRA_ Low Mon_INT_	1.56 (0.74–3.28)	0.24
Low CD8+ T_EMRA_ High Mon_INT_	1.01 (0.46–2.16)	0.99
Low CD8+ T_EMRA_ Low Mon_INT_	1.00	
Age	1.03 (1.01–1.06)	0.003*
Gender (Male)	1.28 (0.79–2.08)	0.31
Diabetes	3.26 (1.99–5.33)	< 0.001*
Albumin (g/dL)	1.21 (0.56–2.21)	0.62
hs-CRP (mg/dL)	1.49 (1.17–1.89)	0.001*
Hemoglobin (g/dL)	1.10 (0.93–1.30)	0.28
Variables in model (independent variable: CVD)		
Immunosenescence		
High CD8+ T_EMRA_ High Mon_INT_	2.39 (1.21–4.70)	0.012*
High CD8+ T_EMRA_ Low Mon_INT_	1.93 (0.97–3.84)	0.06
Low CD8+ T_EMRA_ High Mon_INT_	1.47 (0.72–2.97)	0.29
Low CD8+ T_EMRA_ Low Mon_INT_	1.00	
Age	1.03 (1.01–1.06)	0.001*
Gender (Male)	1.28 (0.81–2.01)	0.29
Diabetes	2.92 (1.86–4.60)	< 0.001*
Albumin (g/dL)	1.03 (0.51–2.08)	0.93
hs-CRP (mg/dL)	1.40 (1.11–1.77)	0.005*
Hemoglobin (g/dL)	1.04 (0.90–1.22)	0.54

Multivariable-adjusted logistic regression models were adjusted for: age, gender, albumin, hemoglobin, DM, hs-CRP and immunophenotype group. The immunophenotype groups were constructed as a categorical variable based on the median-split of the absolute number of CD8+ T_EMRA_ cells and intermediate monocyte number (Mon_INT_), with the Low Mon_INT_ Low CD8+ T_EMRA_ group as the reference group. The results were expressed as odds ratio (OR), 95% confidence interval (CI)

*P value < 0.05

### Uremic toxin *p*-cresyl sulfate positively correlated with levels of CD8+ T_EMRA_ cells

In renal failure patients, retention of uremic toxins is a key mechanism underlying the generation of oxidative stress and inflammation [27]. Others and our previous study also indicated that higher levels of uremic toxins in ESRD patients were related to atherosclerotic complications and mortality [28, 29]. Because aging-related immune changes positively associated with dialysis vintage, we were curious about the relationships between uremic toxins with the level of CD8+ T_EMRA_ and intermediate monocytes. We measured two major uremic toxins, *p*-cresyl sulfate and indoxyl sulfate, in 100 iESRD participants. As shown in Table 5, we found that levels of uremic toxin *p*-cresyl sulfate significantly correlated with higher levels of CD8+ T_EMRA_ cells in both relative percentage and absolute cell number terms. Nevertheless, levels of indoxyl sulfate were not associated with the accumulation of CD8+ T_EMRA_ cells, and levels of uremic toxin were not associated with levels of intermediate monocytes (data not shown).

**Table 5:** Correlations between uremic toxin levels with levels of CD8+ T_EMRA_ cells

Cell subset	*p*-cresyl sulfate (μg/ml)		Indoxyl sulfate (μg/ml)	
	Correlation Coeff.	*P* value	Correlation Coeff.	*P* value
CD8+ T_EMRA (percent CD8+)_	0.22	0.027*	−0.01	0.97
CD8+ T_EMRA (cell number)_	0.22	0.029*	−0.02	0.83

*P value < 0.05

Spearman’s correlation test was performed to analyze the relationships between T_EMRA_ cell and uremic toxin levels. Positive relationships were found between *p*-cresyl sulfate and CD8+ T_EMRA_ cells

## Discussion

The immunity in ESRD, or “iESRD study” was designed with the goal of identifying biomarkers that can accurately assess the health status of ESRD patients undergoing hemodialysis and of investigating the potential mechanism underlying the aggravated aging-related immune changes that may ultimately also apply to the general population. A longitudinal follow-up of the cohort participants is currently being performed to analyze if immune status can predict patients’ survival and if these immune changes will evolve over-time.

The baseline analysis found both adaptive and innate immune subset distribution dramatically changed in ESRD patients compared with healthy individuals. Aging-related changes of lymphocytes and monocytes also positively associate with dialysis vintage and other cardiovascular risk factors in ESRD patients. In the current study, we identified the positive association between these changes and systemic inflammation, and identified a combinatorial aging-related immunophenotype is associated with prevalent atherosclerotic cardiovascular disease in ESRD independently from systemic inflammation. The odds ratio of patients with the high CD8+ T_EMRA_ and high intermediate monocyte immunophenotype for CAD and CVD is higher than every 1 mg/dL increase in high-sensitivity CRP and is in a range close to diabetes. Thus, these findings suggest that aggravated immunosenescence significantly impacts on ESRD patients’ health.

Our study found both CD4+ and CD8+ T cells differentiations are dramatically enhanced in ESRD patients. Compared to healthy donors, ESRD patients have much fewer naïve T cells but at the same time, higher percentage of memory T cells with advanced differentiation-especially CD8+ T_EMRA_ cells. Overall, enhanced immunosenescence is more evident in CD8+ T cells than CD4+ T cells. Consistent with most published studies, CD4+ T cells tend to be less affected by aging than CD8+ T cells [30]. Compared to CD8+ T cells, naïve CD4+ T cells maintain their absolute cell numbers and memory CD4+ T cells maintain a highly diverse T cell receptor repertoire without significant clonal expansion during aging. However, as recently reviewed by Goronzy et al. [31], it remains largely unknown why CD4+ T cells are less susceptible to aging. While a decrease in naïve T cells potentially affects an individual’s response toward new infections and vaccinations [30], memory T cells expressing cytotoxic or terminal differentiation features are increasingly implicated in the pathogenesis of atherosclerotic disease and inflammation [26, 32] although many studies are observational so far. For example, unstable atherosclerotic plaques show a 10-fold increase in their T cell content [33]. In patients with chronic kidney disease without dialysis, CD8+ CD57+ T cell (similar to T_EMRA_ cell) fraction positively associates with arterial stiffness [34]. Terminally differentiated T cells are highly proinflammatory and may produce multiple cytokines [35]. In addition, T_EM_ and T_EMRA_ cells express high level of CX3CR1, a chemokine receptor allows T cells to bind to activated endothelial cells through fractalkine [36, 37] and subsequently cause endothelial injury. Recently, an interesting study [38] demonstrated less immunosenescence in ESRD patients received peritoneal dialysis when compared to hemodialysis. Surprisingly, patients received peritoneal dialysis had more acute rejection events after renal transplantation. As a result, accelerated immunosenescence might be harmful for cardiovascular health in dialysis patients but after transplantation it might be associated with better graft survival.

Our study also found that intermediate and non-classical monocytes are both significantly increased in ESRD. These monocytes are key players in atherosclerosis and previous studies have provided ample evidence of their significant predictive value for CAD and CVD in both the general population as well as renal failure [39–41]. Intermediate monocytes exhibit senescence features because they have shorter telomere compared to classical monocytes and have higher expression of β-galactosidase [42]. Similar to terminally differentiated T cells, intermediate monocytes express both high levels of CCR2 and CX3CR1 [43] and thus are preferentially recruited to the vascular endothelium. In our analysis, ESRD patients exhibit a dramatic increase in these cells when compared to the healthy individuals. Patients with longer dialysis vintage also exhibit higher percentage of intermediate monocytes in their blood.

In humans, CMV virus infection is an important driver of T cell senescence [44] and we have recently found that level of CMV-IgG also positively associated with advanced differentiation of T cells in ESRD patients [45]. Since the iESRD participants are more than 99% CMV seropositive, the enhanced aging-related immune changes we observed is not due to CMV infection per se; but host factors might have modulated CMV-specific immunity. By correlation analyses, dialysis vintage was associated with both T_EMRA_ cells and intermediate monocytes independent of age. The result strongly supports the hypothesis that the duration of renal failure (thus dialysis vintage) may determine the degree of immunosenescence. In addition, there was a statistically significant association between uremic toxin *p*-cresyl sulfate levels with CD8+ T_EMRA_ cells. Although uremic toxin levels did not correlate with monocyte differentiation, one explanation is that one-time, cross-sectional measurement of uremic toxins may not completely capture the complete exposure of uremic milieu. It is important to recognize that the immunosenescent T cell phenotype also does not normalize after successful renal transplantation despite a rapid reduction of uremic toxins [46]. As a result, effects of uremic toxin on immunosenescence might not be reversible by reducing uremic toxin levels.

Our study has several limitations. First, because this is a cross-sectional observational study, the causality between aging-related immune changes and cardiovascular disease cannot be established. Secondly, since the study population is composed of 99% CMV seropositive Taiwanese, it is not known if the findings can be extrapolated to CMV seronegative ESRD patients and to other racial groups. Finally, T cells and monocytes may exhibit aging-related changes in their effector functions that are not reflected by phenotypic changes. In addition, because regulatory T cell differentiation is also affected by renal failure [47], an important direction of further research is to investigate the effects of uremia on regulatory T cells and effector T cells separately.

## Conclusions

ESRD patients exhibit accelerated immunosenescence in both T lymphocyte and monocyte compartment and these changes are positively related to inflammation and cardiovascular morbidities. Chronic exposure to the uremic milieu may directly contribute to these immune changes. ESRD may be used as a disease model in the future for investigating how immunosenescence mediates inflammation and vascular health.

## Additional file

Additional file 1: **Table S1.** Relationships between dialysis vintage and immune cell subsets using tertiles of dialysis vintage, least squares regression and robust regression. **Table S2.** Numbers of age-related immune cells correlate with cardiovascular risk factors and systemic inflammation in ESRD patients. **Table S3.** Comparisons of circulatory T cell and monocyte subset cell numbers between ESRD patients with and without diabetes. **Table S4.** End-stage renal disease patients with concurrent coronary artery disease or cardiovascular disease display more immunosenescence. **Table S5.** Logistic regression model for coronary artery disease and cardiovascular disease using subset percentage to characterize the combinatorial immunophenotype. **Figure S1.** Representative staining of lymphocytes and monocytes. **Figure S2.** Correlogram of immune cell subsets among healthy donors and ESRD patients. (DOCX 1447 kb)

## Abbreviations

CAD: Coronary artery disease
CMV: Cytomegalovirus
CRP: C-reactive protein
CVD: Cardiovascular disease
ESRD: End-stage renal disease
