# Supplementary material for: A comprehensive characterization of aggravated aging-related changes in T lymphocytes and monocytes in end-stage renal disease: the iESRD study
Source: Immun Ageing. 2018 Nov 8;15:27. doi: 10.1186/s12979-018-0131-x (PMC6223078; doi:10.1186/s12979-018-0131-x)
Supplement: Supplementary file 1 — Table S1. Relationships between dialysis vintage and immune cell subsets using tertiles of dialysis vintage, least squares regression and robust regression. Table S2. Numbers of age-related immune cells correlate with cardiovascular risk factors and systemic inflammation in ESRD patients. Table S3. Comparisons of circulatory T cell and monocyte subset cell numbers between ESRD patients with and without diabetes. Table S4. End-stage renal disease patients with concurrent coronary artery disease or cardiovascular disease display more immunosenescence. Table S5. Logistic regression model for coronary artery disease and cardiovascular disease using subset percentage to characterize the combinatorial immunophenotype. Figure S1. Representative staining of lymphocytes and monocytes. Figure S2. Correlogram of immune cell subsets among healthy donors and ESRD patients. (DOCX 1447 kb) [file 12979_2018_131_MOESM1_ESM.docx]

**Additional file 1**

**A comprehensive characterization of aggravated aging-related changes in T lymphocytes and monocytes in end-stage renal disease**

Yen-Ling Chiu*^1-3^, Kai-Hsiang Shu^1,4^, Feng-Jung Yang^2,5^, Tzu-Ying Chou^1^, Ping-Min Chen^1^, Fang-Yun Lay^1^, Szu-Yu Pan^1^, Cheng-Jui Lin^6^, Nicolle Litjens^7^, Michiel Betjes^7^, Selma Bermudez^8^, Kung-Chi Kao^4^, Jean-San Chia^4^, George Wang^9^, Yu-Sen Peng^1^, Yi-Fang Chuang*^8,10^

^1^Division of Nephrology, Department of Medicine, Far Eastern Memorial Hospital, Taipei, Taiwan

^2^Graduate Institute of Clinical Medicine, National Taiwan University College of Medicine, Taipei, Taiwan

^3^Graduate School of Biomedical Informatics, Yuan Ze University, Taoyuan, Taiwan

^4^Graduate Institute of Immunology, National Taiwan University College of Medicine, Taipei, Taiwan

^5^Department of Medicine, National Taiwan University Hospital Yun Lin Branch, Douliu, Taiwan

^6^Division of Nephrology, Department of Internal Medicine, Mackay Memorial Hospital, Taipei, Taiwan

^7^Department of Internal Medicine, Nephrology and Transplantation, Erasmus Medical Center, University Medical Center Rotterdam, Rotterdam, Netherlands

^8^International Health Program, National Yang Ming University School of Public Health, Taipei, Taiwan

^9^ Biology of Healthy Aging Program, Division of Geriatric Medicine and Gerontology, Johns Hopkins University School of Medicine, Baltimore, Maryland, USA

^10^Department of Epidemiology, National Yang Ming University School of Public Health, Taipei, Taiwan

*Corresponding author: Yi-Fang Chuang, M.D., Ph.D.

**Table S1 Relationships between dialysis vintage and immune cell subsets using tertiles of dialysis vintage, least squares regression and robust regression**

| *Cell Subset* | *Dialysis vintage (tertiles)*  *β* | | | *P for trend* | *Dialysis vintage (least squares regression)*  *β* | *P value* | *Dialysis vintage (robust regression)*  *β* | *P value* |
| --- | --- | --- | --- | --- | --- | --- | --- | --- |
|  | *> 7.6 years* | *3.4-7.6 years* | *< 3.4 years* |  |  |  |  |  |
| CD4+ T cells | | | | | | | | |
| Central memory T cells | -6.3 | -4.2 | Ref. | <0.001* | -0.6 | <0.001* | -0.61 | <0.001* |
| Effector Memory T cells | 3.1 | 2.3 | Ref. | 0.044* | 0.41 | 0.001* | 0.25 | 0.031* |
| CD8+ T cells | | | | | | | | |
| Effector Memory T cells | -8.4 | -4.0 | Ref. | <0.001* | -0.67 | <0.001* | -0.68 | <0.001* |
| Terminally differentiated T cells | 5.4 | 2.2 | Ref. | 0.004* | 0.47 | 0.002* | 0.52 | 0.001* |
| Monocytes | | | | | | | | |
| Classical monocytes | -4.9 | -3.7 | Ref. | 0.001* | -0.32 | 0.005* | -0.35 | 0.003* |
| Intermediate monocytes | 1.6 | 0.4 | Ref. | 0.046* | 0.27 | <0.001* | 0.20 | <0.001* |

To further validate the effects of dialysis vintage on immune changes, ESRD patients were separated into tertiles for P for trend analysis. Relationships were also further analyzed by robust regression to eliminate the concern of outliers. *: P value < 0.05.

- **Table S2 Numbers of age-related immune cells correlate with cardiovascular risk factors and systemic inflammation in ESRD patients**

| Cell numbers | CD8+ T_NAIVE_ | | - CD8+ T_EMRA_ | | Intermediate Monocyte | |
| --- | --- | --- | --- | --- | --- | --- |
|  | Correlation Coeff. | P value | Correlation Coeff. | P value | Correlation Coeff. | P value |
| Hemoglobin (g/dL) | 0.37 | <0.001* | 0.04 | 0.37 | 0.03 | 0.46 |
| Albumin (g/dL) | 0.44 | <0.001* | 0.04 | 0.47 | 0.05 | 0.28 |
| Ca×P  (mg^2^/dL^2^) | 0.11 | 0.03* | 0.001 | 0.98 | 0.06 | 0.24 |
| Uric acid (mg/dL) | 0.12 | 0.04* | -0.04 | 0.52 | 0.08 | 0.28 |
| Cholesterol (mg/dL) | 0.19 | <0.001* | -0.002 | 0.95 | 0.23 | <0.001* |
| Triglyceride (mg/dL) | 0.11 | 0.04* | 0.07 | 0.18 | 0.24 | <0.001* |
| CT ratio | -0.17 | 0.019* | -0.02 | 0.79 | 0.13 | 0.07 |
| i-PTH  (pg/mL) | 0.01 | 0.84 | -0.05 | 0.37 | -0.04 | 0.41 |
| Ferritin  (ng/mL) | -0.06 | 0.21 | 0.07 | 0.14 | 0.08 | 0.12 |
| hs-CRP  (mg/dL) | -0.13 | 0.01* | 0.07 | 0.15 | 0.34 | <0.001* |

Spearman’s correlation was performed to analyze the relationships between immune cell numbers and other potential cardiovascular risk factors. *: P value < 0.05.

**Table S3 Comparisons of circulatory T cell and monocyte subset cell numbers between ESRD patients with and without diabetes**

| - Absolute cell number | - DM (184) | - Non-DM (228) | - P value |
| --- | --- | --- | --- |
| - CD4+ T cells |  |  |  |
| - Naïve T cells | - 112.7 | - 105.9 | - 0.5 |
| - Central Memory T cells | - 160.9 | - 143.3 | - 0.088 |
| - Effector Memory T cells | - 105.9 | - 97.5 | - 0.28 |
| - Terminally Differentiated T cells | - 9.7 | - 7.3 | - 0.044* |
| - CD8+ T cells |  |  |  |
| - Naïve T cells | - 46.3 | - 39.2 | - 0.165 |
| - Central Memory T cells | - 15.2 | - 12.2 | - 0.11 |
| - Effector Memory T cells | - 69.4 | - 57.7 | - 0.049* |
| - Terminally Differentiated T cells | - 78.0 | - 78.0 | - 0.994 |
| - Monocytes |  |  |  |
| - Classical Monocytes | - 235.6 | - 217.3 | - 0.117 |
| - Intermediate Monocytes | - 45.2 | - 39.7 | - 0.143 |
| - Non-Classical Monocytes | - 81.2 | - 75.3 | - 0.238 |

Absolute cell numbers (per μl blood) of naïve (T_NAIVE_), stem cell memory (T_SCM_), central memory (T_CM_), effector memory (T_EM_), terminally differentiated (T_EMRA_) subsets and three monocyte subsets (classical monocytes, intermediate monocytes, non-classical monocytes) were compared between ESRD patients with and without diabetes. The inter-group differences were analyzed by Student’s t-test. *: P value < 0.05.

**Table S4 End-stage renal disease patients with concurrent coronary artery disease or cardiovascular disease display more immunosenescence**

| - Cell subset - percentage | - Coronary artery disease - (CAD) | | | | - Cardiovascular disease - (CVD) | | |
| --- | --- | --- | --- | --- | --- | --- | --- |
|  | - CAD (n=106) | - No CAD (n=306) | | - P value | - CVD (n=132) | - No CVD (n=280) | - P value |
| - CD8+ T_NAIVE_ | - 18.3 (13.3) | - 22.9 (16.8) | | - 0.011* | - 19.0 (14.0) | - 23.1 (16.8) | - 0.017* |
| - CD8+ T_EMRA_ | - 43.0 (16.7) | - 36.3 (16.4) | | - <0.001* | - 42.0 (16.3) | - 36.2 (16.6) | - <0.001* |
| - Intermediate Monocyte | - 10.8 (6.3) | - 9.9 (6.6) | | - 0.24 | - 11.1 (6.6) | - 9.7 (6.5) | - 0.044* |
| - *Absolute cell* - *number* | - *Coronary artery disease* - *(CAD)* | | | | - *Cardiovascular disease* - *(CVD)* | | |
|  | - CAD (n=106) | - No CAD (n=306) | - P value | | - CVD (n=132) | - No CVD (n=280) | - P value |
| - CD8+ T_NAIVE_ | - 48.6 (47.4) | - 56.6 (66.1) | - 0.09 | | - 49.6 (49.5) | - 56.8 (66.9) | - 0.14 |
| - CD8+ T_EMRA_ | - 119 (90.6) | - 101 (96.6) | - 0.72 | | - 119 (90.4) | - 99.5 (96.9) | - 0.86 |
| - Intermediate Monocyte | - 50.0 (36.9) | - 38.1 (33.2) | - 0.17 | | - 46.1 (34.1) | - 37.6 (33.3) | - 0.21 |

- Percentages and absolute numbers of circulatory immune cell percentages including CD8+ naïve, CD8+ T_EMRA_ and intermediate monocyte were compared between patients with and without coronary artery disease (CAD). Patients with CAD or CVD showed higher percentages of CD8+ T_EMRA_ cells and CD14++CD16+ intermediate monocytes in their peripheral blood. *: P value < 0.05.

**Table S5 Logistic regression model for coronary artery disease and cardiovascular disease using subset percentage to characterize the combinatorial immunophenotype**

| - Variables in model - (independent variable: CAD) | OR (95% CI) | - P value |
| --- | --- | --- |
| - Immunophenotype |  |  |
| - High CD8+ T_EMRA_ High Mon_INT_ | - 2.29 (1.17-4.47) | - 0.016* |
| - High CD8+ T_EMRA_ Low Mon_INT_ | - 1.41 (0.69-2.91) | - 0.34 |
| - Low CD8+ T_EMRA_ High Mon_INT_ | - 0.82 (0.38-1.75) | - 0.62 |
| - Low CD8+ T_EMRA_ Low Mon_INT_ | - 1.00 |  |
| - Age | - 1.03 (1.01-1.05) | - 0.01* |
| - Gender (Male) | - 1.24 (0.77-2.03) | - 0.37 |
| - Diabetes | - 3.56 (2.16-5.86) | - <0.001* |
| - Albumin (g/dL) | - 1.21 (0.51-2.06) | - 0.62 |
| - hs-CRP (mg/dL) | - 1.50 (1.18-1.90) | - 0.001* |
| - Hemoglobin (g/dL) | - 1.12 (0.95-1.32) | - 0.18 |
|  |  |  |
| - *Variables in model* - *(independent variable: CVD)* | - *OR (95% CI)* | - *P value* |
| - Immunophenotype |  |  |
| - High CD8+ T_EMRA_ High Mon_INT_ | - 2.32 (1.24-4.35) | - 0.008* |
| - High CD8+ T_EMRA_ Low Mon_INT_ | - 1.23 (0.63-2.41) | - 0.54 |
| - Low CD8+ T_EMRA_ High Mon_INT_ | - 0.97 (0.49-1.93) | - 0.93 |
| - Low CD8+ T_EMRA_ Low Mon_INT_ | - 1.00 |  |
| - Age | - 1.03 (1.01-1.05) | - 0.001* |
| - Gender (Male) | - 1.25 (0.79-1.96) | - 0.34 |
| - Diabetes | - 3.22 (2.03-5.12) | - <0.001* |
| - Albumin (g/dL) | - 1.11 (0.55-2.23) | - 0.78 |
| - hs-CRP (mg/dL) | - 1.40 (1.11-1.77) | - 0.004* |
| - Hemoglobin (g/dL) | - 1.07 (0.92-1.24) | - 0.40 |

Multivariable-adjusted logistic regression models were adjusted for: age, gender, albumin, hemoglobin, DM, hs-CRP and immunophenotype group. The immunophenotype groups were constructed as a categorical variable based on the median-split of the absolute number of CD8+ T_EMRA_ cells and intermediate monocyte number (Mon_INT_), with the Low Mon_INT_ Low CD8+ T_EMRA_ group as the reference group. The results were expressed as odds ratio (OR), 95% confidence interval (CI). *: P value < 0.05.

**Figure S1 Representative staining of lymphocytes and monocytes**


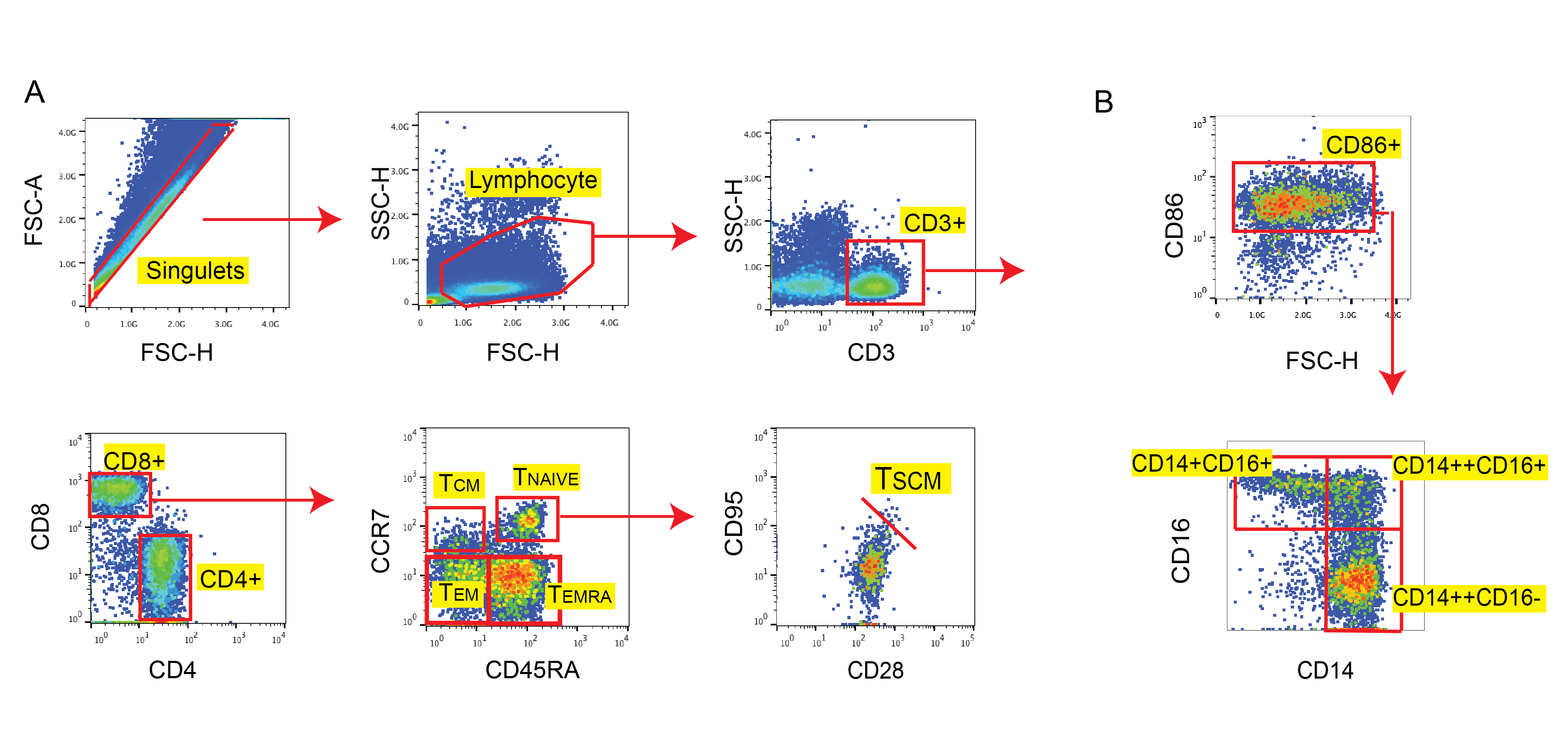


(A) Representative figures of the gating strategies for T cell differentiation panel. First, forward scatter height (FSC-H) and area (FSC-A) were used to gate on singlets. Lymphocytes were selected by their forward (FSC-H) and side scatter (SSC-H) characteristics, and further classified into CD3+CD4+ and CD3+CD8+ T cells. These populations were further divided into TNaïve (TNaive), central memory (TCM), effector memory (TEM) and terminally differentiated effector memory CD45RA+ cells (TEMRA) according to surface expression of CCR7 and CD45RA. The CD28+CD95+ stem-cell memory T cells (TSCM) were further defined in the TNaive subset. (B) Representative figures of the gating strategies for monocyte panel. After singulet and FSC-H/SSC-H gating, monocytes were additionally gated by CD86 expression, and then classified into classical (CD14++CD16-), intermediate (CD14++CD16+), and non-classical (CD14+CD16++) monocyte subsets.

**Figure S2 Correlogram of immune cell subsets among healthy donors and ESRD patients**

**
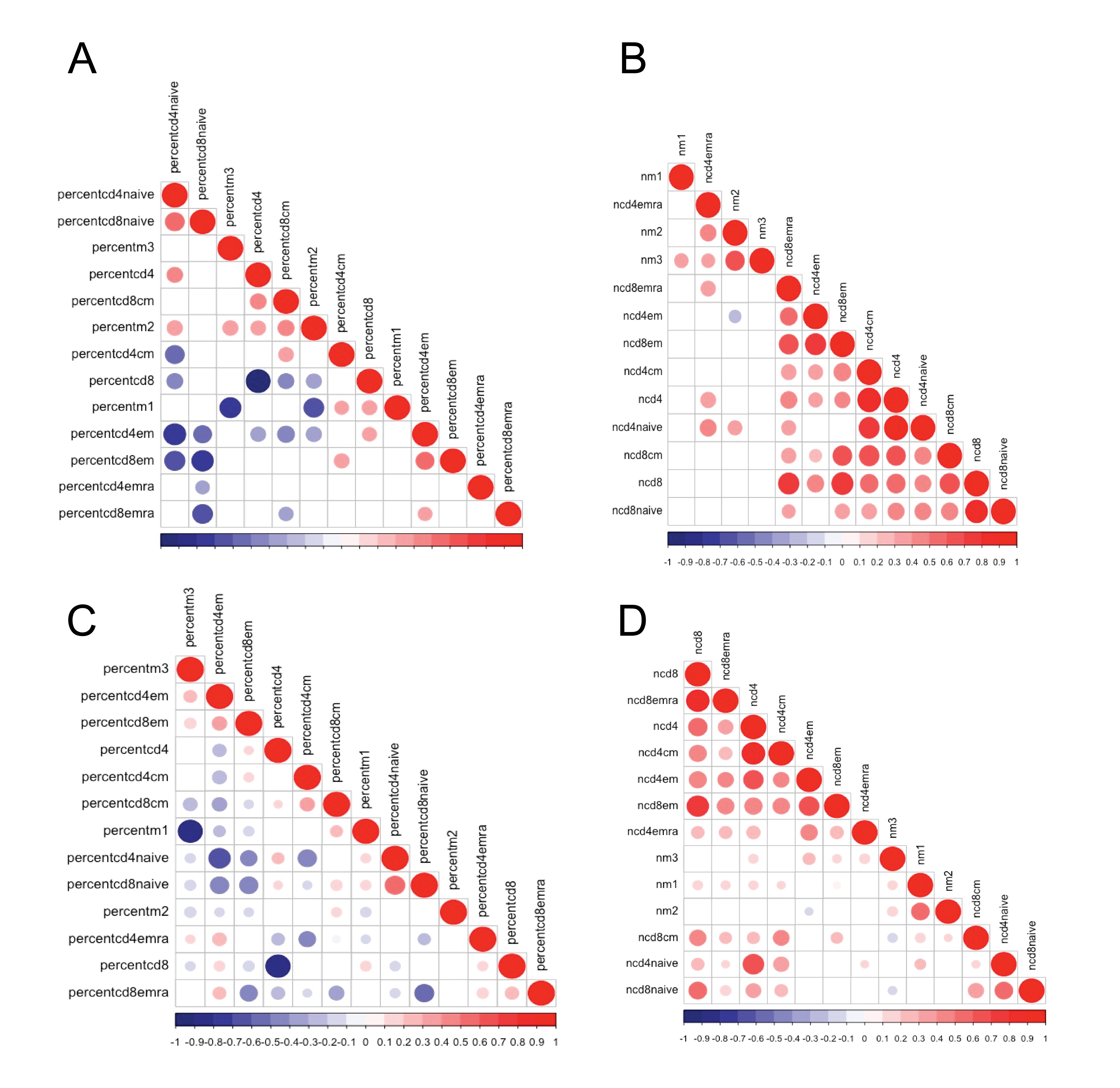
**

- Percentages as well as absolute number of immune cell subsets were analyzed in the correlation matrix performed by the R corrplot package. (A): Correlations among healthy donors based on cell type percentage; (B) Correlations among healthy donors based on absolute cell number; (C) Correlations among ESRD patients based on cell type percentage; (D) Correlations among ESRD patients based on absolute cell number. Only statistically significant associations are shown. Both intensity of circle color and circle size indicate the strength of association (correlation coefficient). Arrangements of cell subsets are based on hierarchial clustering. Abbreviations for monocyte subsets: m1: classical; m2: intermediate; m3: non-classical.
